# Plasma cell leukemia

**DOI:** 10.1590/S1679-45082013000100025

**Published:** 2013

**Authors:** Clarissa Lima e Moura de Souza, Guilherme Fleury Perini, Nelson Hamerschlak, Paulo Augusto Achucarro Silveira

**Affiliations:** 1 Hospital Israelita Albert Einstein, São Paulo, SP, Brazil

A 74-year-old woman with renal failure and lytic lesions in the spine was admitted in our hospital. She was diagnosed with IgG kappa multiple myeloma. In addition, monosomy of chromosome 13 and IgH infusion by FISH were seen.

The patient underwent five cycles of VMP (bortezomib, melphalan and prednisone) that achieved complete response, followed by maintenance with bortezomib. However, 18 months later, the patient was readmitted with asthenia and leucocytosis. Atypical cells with plasmacytoid characteristics were detected in peripheral blood smears, consistent with plasma cell leukemia (PCL) (Figures 1 and 2).

**Figure 1:**
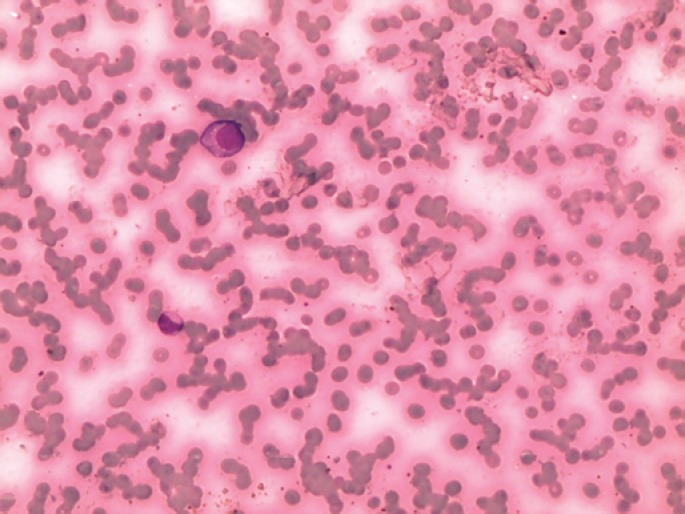
Plasma cell in peripheral blood

**Figure 2:**
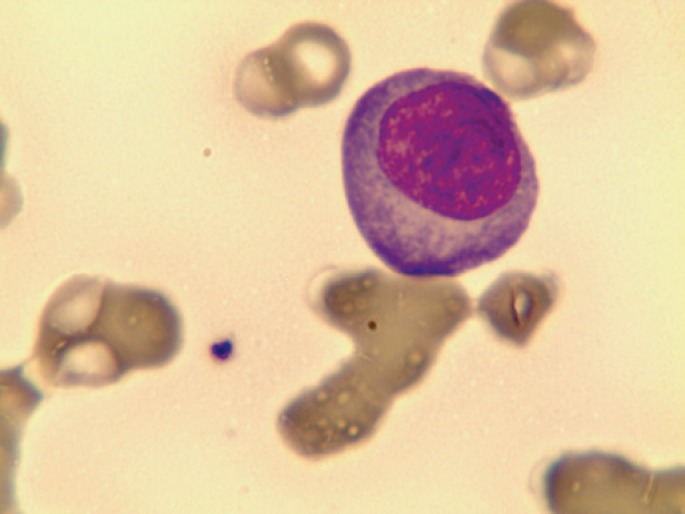
Plasma cell

PCL is a rare lymphoproliferative disorder featured by the presence of more than 20% of plasma cells in peripheral blood^(1)^. This disease can be primary, but in 40% of cases there is the diagnosis of multiple myeloma. Morphologically, plasma cells are oval-shaped with large basophilic cytoplasm, round eccentric nucleus and spoke wheel appearance of chromatin without nucleolus. More immature forms can present dispersed chromatin, prominent nucleoli and high nucleocytoplasmic relationship.

Prognosis of PCL patients is poor, and the mean survival is 18–20 months mainly in secondary disease cases in which cytogenetic changes of poor prognosis with del (17p) and p53 loss are often found^(2)^.

The patient underwent rescue chemotherapy, without response. After that, her family opted for palliative treatment.

## References

[1] McKenna RW, Kyle RA, Kuehl WM, Gtogan TM, Harris NL, Coupland RW, Swerdlow SH, Campo E, Harris NL, Jaffe ES, Pileri SA, Stein H (2008). Plasma cell neoplasm. World Health Organization classification of tumors of haematopoietic and lymphoid tissues.

[2] García-Sanz R, Orfão A, González M, Tabernero MD, Bladé J, Moro MJ (1999). Primary plasma cell leukemia: clinical, immunophenotypic, DNA ploidy, and cytogenetic characteristics. Blood.

